# Investigating thyroid dysfunction in the context of COVID-19 infection

**DOI:** 10.1016/j.amsu.2022.104806

**Published:** 2022-10-31

**Authors:** Aashna Mehta, Wireko Andrew Awuah, Rohan Yarlagadda, Jacob Kalmanovich, Helen Huang, Mrinmoy Kundu, Esther Patience Nansubuga, Leilani Lopes, Bikona Ghosh, Mohammad Mehedi Hasan

**Affiliations:** a University of Debrecen-Faculty of Medicine, Debrecen, 4032, Hungary; b Sumy State University, Sumy, Ukraine; c Rowan University School of Osteopathic Medicine, Stratford, NJ, USA; d Drexel University, College of Medicine, Philadelphia, PA, USA; e Royal College of Surgeons in Ireland, University of Medicine and Health Science, Dublin, Ireland; f Institute of Medical Sciences and SUM Hospital, Bhubaneswar, India; g Leeds Medical School, University of Leeds, UK; h Western University of Health Sciences, College of Osteopathic Medicine of the Pacific-Northwest, Lebanon, OR, USA; i Dhaka Medical College, Dhaka, Bangladesh; j Department of Biochemistry and Molecular Biology, Faculty of Life Science, Mawlana Bhashani Science and Technology University, Tangail, Bangladesh

## Abstract

COVID-19 is a contagious viral infection caused by severe acute respiratory syndrome coronavirus 2 (Sars-CoV-2). One of the key features of COVID-19 infection is inflammation. There is increasing evidence pointing to an association between cytokine storm and autoimmunity. One autoimmune disease of interest in connection to COVID-19 is hyperthyroidism. COVID-19 has been shown to decrease TSH levels and induce thyrotoxicosis, destructive thyroiditis, and de novo Graves’ disease. It has also been suggested that the immune response against SARS-CoV-2 antigens following vaccination can cross-react through a mechanism called molecular mimicry which can elicit autoimmune reactivity, potentially leading to potential thyroid disease post vaccine. However, if the COVID-19 vaccine is linked to reduced COVID-19 related serious disease, it could potentially play a protective role against post COVID-19 hyperthyroidism (de novo disease and exacerbations). Further studies investigating the complex interplay between COVID-19 or COVID-19 vaccine and thyroid dysfunction can help provide substantial evidence and potential therapeutic targets that can alter prognosis and improve COVID-19 related outcomes in individuals with or without preexisting thyroid disease.

## Highlights

• A careful review of current literature suggests a possible correlation of COVID-19 induced inflammation and hyperthyroidism.
• The association could be possibly explained by molecular mimicry between the proteins of SARS-CoV-2 with those in the thyroid.
• Reports of de novo thyroiditis have emerged post COVID-19 vaccine, but the COVID-19 has not shown to cause exacerbations in preexisting thyroid disease.
• There is a scarcity of literature on the complex interplay between COVID-19 or COVID-19 vaccine and thyroid dysfunction.

## 1 Introduction

Coronavirus disease 2019 (COVID-19) has created a very tumultuous time for the world. COVID-19 is a contagious viral infection caused by severe acute respiratory syndrome coronavirus 2 (Sars-CoV-2). Since the first case in late December 2019, 510,270,667 confirmed cases of COVID-19, including 6,233,526 deaths have been reported to the WHO [1]. Consequently, COVID-19 was declared a pandemic in March 2020 by the WHO and is known as the most consequential global health crisis since the era of the influenza pandemic of 1918.

COVID-19, like other coronaviruses, is a positive-stranded (+ssRNA) virus. SARS-CoV-2 enters the hosts' cells by binding the SARS-CoV-2 spike or S protein (S1) to the ACE2 receptors that are abundantly present on the respiratory epithelium such as type II alveolar epithelial cell [2]. Upon its entry into the host, replication, transcription, and translation of the viral RNA leads to the production of many proteins. Some of these proteins include virally encoded chymotrypsin-like protease (3CLpro) or main protease (Mpro), as well as papain-like proteases for producing 16 non-structural proteins with known or predicted RNA synthesis and modification functions [2]. The pathogenesis of this disease is related to the function of the NSPs (Non-Structural Proteins) and other structural proteins which can block the hosts’ innate immune responses [3].

One of the key features of COVID-19 infection is inflammation. There is increasing evidence pointing to an association between one's immune response, inflammatory markers, and a potential cytokine storm with the progression, severity, and outcome of COVID-19. Studies have shown that a maladaptive immune response, with an increase in innate immune cell lineages and reduction in lymphocytes (lymphopenia), are associated with more severe disease and poorer outcomes [4]. A delayed type of interferon response during response may lead to delayed priming of the more robust adaptive immune response. This can lead to a high viral load and a more severe and destructive immune response as seen in a minority of patients [4]. This overwhelming response to COVID-19 has been linked to a cytokine storm which contributes to some of the more severe pathologies associated with it such as acute respiratory distress syndrome, multi-organ failure, and in particular autoimmune diseases [5,6]. An example of this effect can be seen in the changes that occur in the thyroid function. COVID-19 has been shown to decrease TSH levels and induce thyrotoxicosis, destructive thyroiditis, and de novo Graves' disease [7]. The effects of cytokine storm may also noticeably impact those with pre-existing thyroid function especially, the elderly whose immune system may be less robust with higher risk of thromboembolic complications and mortality [8,9].

**Fig. 1:**
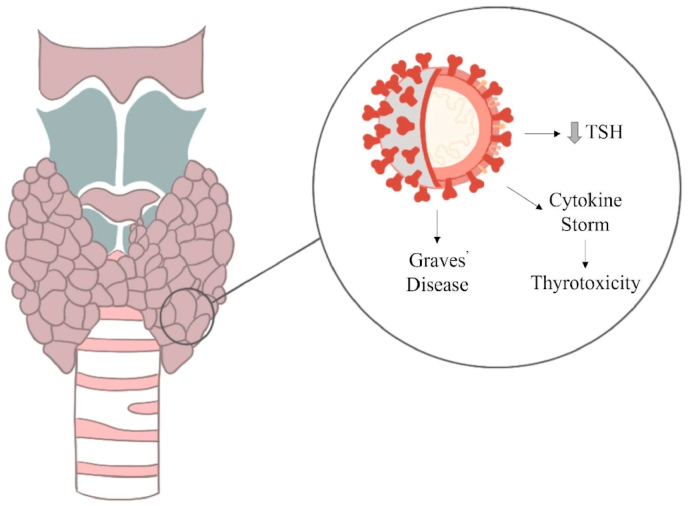
The different mechanisms of action that COVID-19 has been postulated to have on the thyroid. Original figure drawn by LL.

Much research has gone into understanding the pandemic and the effects that it has on both previously healthy patients and those with other comorbidities. This review discusses in detail the inflammatory response by COVID-19, on thyroid function (Fig. 1) in individuals with preexisting hyperthyroidism as well as the development of de novo hyperthyroidism.

## 2 COVID-19 Induced autoimmunity and effect on thyroid function

Autoimmune disease could arise as a complication of COVID-19 infection. This is evidenced by numerous reports that have found a connection with the development of autoimmune diseases after SARS-CoV-2 infection such as cold agglutinin syndrome (CAS) and autoimmune hemolytic anemia [10]. Furthermore, previous studies suggested the presence of various autoantibodies among COVID-19 infected patients in different frequencies: antinuclear antibodies (ANA) in 35.6%, anti-Ro/SSA in 25%, rheumatoid factor in 19%, lupus anticoagulant in 11% and antibodies against interferon (IFN)-I in 10% [11]. These antibodies are associated with multiples autoimmune diseases such as immune-mediated vasculitis, arthritis, idiopathic inflammatory myopathies, and systemic lupus erythematosus.

De novo development of autoimmune disease induced by COVID-19 may complicate the prognostic course for patients with existing autoimmune conditions. During the pandemic, there was a sizable portion of patients with autoimmune diseases who had abandoned their medications, either from fearing the immunosuppressive influence of medications or due to lack of availability [12].

One autoimmune disease of interest in connection to COVID-19 is hyperthyroidism. SARS-CoV-2 uses ACE2 combined with the transmembrane protease serine 2 (TMPRSS2) as the key molecular complex to infect the host cell which are expressed highly in both thyroid gland and lungs [13]. Similar molecular complex could explain the development of numerous thyroid diseases including thyrotoxicosis and Graves' disease which is the most common form of hyperthyroidism [14]. Therefore, COVID-19 infected hyperthyroid patients are at increased risk of induction or relapse of Graves' disease as an autoimmune response [9]. Moreover, cytokine storm produced by a severe SARS-CoV-2 infection may be thyrotoxic in older individuals with poorly managed Graves’ disease, increasing COVID-19-related mortality [15].

## 3 COVID-19 Induced thyrotoxicosis

Association between COVID-19 and thyrotoxicosis has been suggested by several studies. One such study is TGYRCOV study by Lania et al. (2020) that evaluated thyroid function by thyroid function test and serum interleukin-6 (IL-6) among 287 diagnosed COVID-19 patients [8]. Due to the exclusion criteria of this study, participants with no history of thyroid disorder or a subclinical manifestation were included only [8]. 31 participants had developed thyrotoxicosis, whilst 27 with subclinical thyrotoxicosis which was evidenced by symptoms of thyrotoxicosis such as atrial fibrillation, thromboembolic events and higher serum level of free thyroxin [8]. Moreover, different pathways led to overproduction of thyroid hormone that manifested as thyrotoxicosis [16].

This finding was supported by another study where two groups were analyzed: 78 patients infected with COVID-19 and 85 in the control group, both of which did not have a history of thyroid disease [17]. This study evaluated the potential prevalence of thyrotoxicosis, as an indicator of subacute thyroiditis and measured C-reactive protein as well. Results concluded that severely affected COVID-19 patients present with thyrotoxicosis with low serum thyroid-stimulating hormone (TSH) levels on a likely background of subacute thyroiditis [17].

On a similar note, Graves' disease, an autoimmune condition that can cause an overactive thyroid, has been found to be induced by COVID-19 infection [18,19]. A review by Murugan and Alzahrani (2021) analyzed a study of five cases of COVID-19 infection that led to the development of Graves’ disease. All the participants involved were female within the age range of 21–61 years and had been assessed from 30 to 60 days since they had initially been infected with COVID-19 [19]. All the participants had reported previous thyroid disease that had been treated and all the participants had been in clear remission before the study had taken place [19]. This study suggests that COVID-19 may exacerbate underlying existing autoimmune disorders whilst the previously discussed studies shows that COVID-19 is a contributor to new-onset autoimmunity.

### 3.1 Influence of the COVID-19 vaccine on thyroid function: Reports of De novo hyperthyroidism and potential protective role

**Fig. 2:**
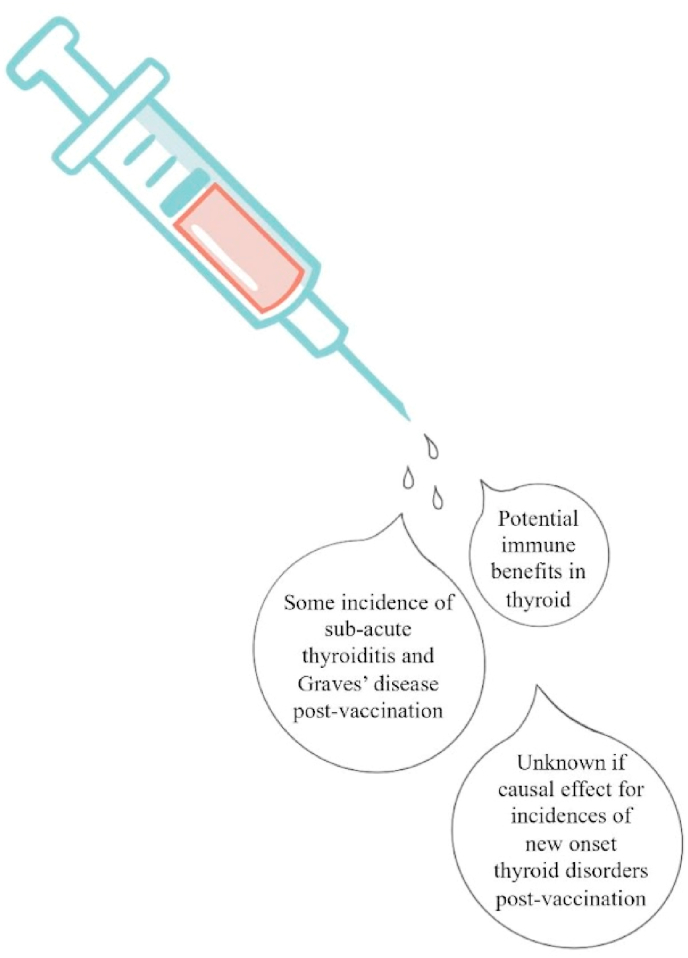
Illustration representing possible influence of COVID-19 vaccine on patients with preexisting thyroid disease.Original figure drawn by LL.

In addition to the risk of developing thyroid disorders following COVID-19 infection, concerns surrounding hyper/hypothyroidism post-SARS-CoV-2 vaccination have become a topic of interest (Fig. 2). Several vaccines have been developed against the SARS-CoV-2 virus, with messenger RNA (mRNA) vaccines by Pfizer-BioNTech and Moderna most widely distributed from the United States [20,21]. Other mRNA vaccines include CureVac from Europe, Janssen-Johnson & Johnson, AstraZeneca, Sputnik-V, and CanSino [21]. However, the mechanism of the vaccine can evoke similar immune responses that are induced by COVID-19 infection [22]. It has been suggested that the immune response against SARS-CoV-2 antigens following vaccination can cross-react through a mechanism called molecular mimicry which can elicit autoimmune reactivity, potentially leading to autoimmune diseases [20]. Generally, the clinical course of Subacute Thyroiditis(SAT) is self-limiting and goes through phases of thyrotoxicosis, hypothyroidism, and eventual euthyroidism, but may require additional symptomatic treatment.

In previous epidemics, thyroiditis development in previously healthy patients was implicated in vaccinations against influenza, HPV, and HBV [22]. Emerging evidence has shown several instances where previously healthy patients with no preceding symptoms of viral infection developed thyroid disorders post-SARS-CoV-2 vaccination, most of which were SAT and, less commonly, Graves’ Disease [22]. A recent review of preliminary evidence reported 83 cases of thyroid dysfunction after vaccination, with mRNA-based vaccines being the most common, followed by viral vector vaccines and inactivated vaccines [23]. Based on vaccine brands, almost half of SAT cases were attributed to Pfizer vaccinations. Subacute thyroiditis, known as de Quervain thyroiditis or granulomatous thyroiditis is caused by viral infections of the upper respiratory tract such as mumps and influenza [24]. Cases of SAT have been presently reported post-COVID-19 infection and SARS-CoV-2 vaccination administration with varying degrees of symptomatic onset [[25], [26], [27], [28]]. A case report detailing eleven patients showed that they experienced symptoms of SAT 22 days after vaccination [29]. In another clinical case study, three patients with no previous history of thyroid disease presented with thyrotoxicosis 10–20 days after receiving the Pfizer, Bio-NTech, or Moderna mRNA vaccine [30]. A literature review conducted by Jhon et al. revealed that out of the 22 SAT cases reported, 13 patients required steroid therapy and the remaining cases subsided with supportive treatment [31].

In rare cases, Graves' Disease was implicated after SARS-CoV-2 vaccination but the pathophysiological mechanism underlying this remains unclear. To date, six reported cases of Graves' Diseases did not exhibit typical symptoms of exophthalmos or dermopathy, with five of these patients having received mRNA vaccines [32,33]. One patient who had not received an mRNA vaccine had a family history of hyperthyroidism and was diagnosed with Graves' Disease 5-days after an adenovirus-vectored SARS-CoV-2 vaccination [34]. The first case study to ever be reported of Graves’ Disease post-vaccination presented with two patients who developed thyroid hyperactivity based on thyroid function tests three days after their immunizations [35]. The other three case studies reported hyperthyroidism based on Tc99 scintigraphy accumulation in patients that have had a long-standing history of thyroid dysfunction before their vaccination [33,36].

Despite cases reporting new-onset autoimmune disorders after being vaccinated against COVID-19, future direction of research should consider the possibility of SARS-CoV-2 vaccination protecting patients from post-covid subacute thyroiditis or Graves’ Disease. The mechanism of the vaccine provides protection via SARS-CoV-2 antibodies, triggering adaptive immune responses and stimulating a hyperinflammatory reaction [22]. SARS-CoV-2 vaccination induces an effective and strong immune response that is responsible for decreasing the risk of serious disease from COVID-19 [21]. Moreover, there are reported immune benefits of SARS-CoV-2 vaccination, with proven efficacy in protecting children from coronavirus-related multisystem inflammatory syndrome [37]. Post-vaccination individuals tend to exhibit elevated expressions of type 1 interferon (IFN) and anti-SARS-CoV-neutralizing antibody production [38]. As such, cases of autoimmune flares could be potentially seen as mere side effects from inflammatory cytokine bursts of IFN that would transiently resolve overtime. This could potentially explain why patients who developed hyperthyroidism or hypothyroidism post-vaccination did not experience severe complications and experienced symptomatic relief with supportive care or steroids [31]. Regardless, it is still widely unknown whether the onset of autoimmune thyroid disorders is a causal association with SARS-CoV-2 vaccination or a mere coincidence. Currently, there is a lack of evidence to suggest the protective influence of SARS-CoV-2 vaccination against autoimmune conditions of the thyroid [38]. However, future studies should focus on investigating the possibility of the vaccine preventing autoimmune flares and thyroid disorders post-COVID infection. To further elucidate the potential immune benefits of SARS-CoV-2 vaccination against thyroid disorders, further longitudinal studies should compare rates of thyroid disorders based on SARS-CoV-2 vaccination status and conduct a comparative conclusion based on the results.

### 3.2 The role of vaccine in altering COVID 19 prognosis in patients with preexisting thyroid disease

As implicated thus far, COVID-19 infection has been recently associated with thyroid dysfunction [39]. This can be due to direct destruction of the thyroid gland by SARS-CoV-2 virus, sick euthyroid syndrome and immune mediated mechanisms [39]. In rare cases thyroid disorders have also been reported after receiving the COVID19 vaccine [40]. One such case was a 42-year-old female with no past medical history of thyroid related disease who was diagnosed as such after receiving the first dose of the Pfizer/BioNTech mRNA vaccine for COVID-19 [27]. It might be possible that SARS-CoV-2 proteins in the SARS-CoV-2 vaccines cross-react with thyroid target proteins due to molecular mimicry to cause various thyroid disorders such as subacute thyroiditis [41].

However, the COVID19 vaccine is not contraindicated in patients with pre-existing thyroid diseases like Hashimoto's thyroiditis and Graves' disease. The American Thyroid Association and European Thyroid Association reported that if patients with thyroid disorders such as autoimmune thyroid disease and thyroid cancer are medically stable, they should still receive the COVID-19 vaccination [42]. If the patient is medically unstable, then the physician will decide on a case-by-case basis [33].

## 4 Conclusion

A careful review of current literature suggests a possible correlation of COVID-19 induced inflammation and hyperthyroidism. This association could be explained by the molecular mimicry between the proteins of SARS-CoV-2 with those in the thyroid. There is a scarcity of literature on a possible preventive role the SARS-CoV-2 vaccination against the development of autoimmune thyroid disorders. However, the COVID-19 vaccine has not been observed to cause exacerbations with preexisting thyroid disease and the benefits outweigh the risks. Even patients who developed hypo/hyperthyroidism post-COVID vaccine did not experience severe complications and received symptomatic relief with steroids and supportive care. Further studies investigating the complex interplay between COVID-19 and thyroid dysfunction can help provide substantial evidence and potential therapeutic targets that can alter prognosis and improve COVID-19 related outcomes in individuals with or without preexisting thyroid disease.

## Ethical approval

N/A.

## Source of funding

N/A.

## Author contribution

Substantial contribution to the Conception and design of the work: All authors under the guidance of Aashna Mehta and Wireko Andrew Awuah. Drafting the work and critical revision: All authors under the guidance of Aashna Mehta, Wireko Andrew Awuah, and Rohan Yarlagadda, and Mohammad Mehedi Hasan. All the authors read and approved the final version of the manuscript.

## Trail registry number

N/A.

## Garantor

Mohammad Mehedi Hasan, Department of Biochemistry and Molecular Biology, Faculty of Life Science, Mawlana Bhashani Science and Technology University, Tangail, Bangladesh; mehedi.bmb.mbstu@gmail.com (MMH).

## Provenance and peer review

Not commissioned, externally peer reviewed.

## Data availability statement

No data available.

## Declaration of competing interest

The authors declare that they have no known competing financial interests or personal relationships that could have appeared to influence the work reported in this paper.
